# Lipin-1 regulates cancer cell phenotype and is a potential target to potentiate rapamycin treatment

**DOI:** 10.18632/oncotarget.3595

**Published:** 2015-03-14

**Authors:** Laura Brohée, Stéphane Demine, Jérome Willems, Thierry Arnould, Alain C. Colige, Christophe F. Deroanne

**Affiliations:** ^1^ Laboratory of Connective Tissues Biology, GIGA-Cancer, University of Liège, Tour de Pathologie, Sart-Tilman, Belgium; ^2^ Laboratory of Biochemistry and Cell Biology (URBC), NARILIS (Namur Research Institute for Life Sciences), University of Namur (UNamur), Namur, Belgium

**Keywords:** lipin-1, prostate cancer, RhoA, metabolism, rapamycin

## Abstract

Lipogenesis inhibition was reported to induce apoptosis and repress proliferation of cancer cells while barely affecting normal cells. Lipins exhibit dual function as enzymes catalyzing the dephosphorylation of phosphatidic acid to diacylglycerol and as co-transcriptional regulators. Thus, they are able to regulate lipid homeostasis at several nodal points. Here, we show that lipin-1 is up-regulated in several cancer cell lines and overexpressed in 50 % of high grade prostate cancers. The proliferation of prostate and breast cancer cells, but not of non-tumorigenic cells, was repressed upon lipin-1 knock-down. Lipin-1 depletion also decreased cancer cell migration through RhoA activation. Lipin-1 silencing did not significantly affect global lipid synthesis but enhanced the cellular concentration of phosphatidic acid. In parallel, autophagy was induced while AKT and ribosomal protein S6 phosphorylation were repressed. We also observed a compensatory regulation between lipin-1 and lipin-2 and demonstrated that their co-silencing aggravates the phenotype induced by lipin-1 silencing alone. Most interestingly, lipin-1 depletion or lipins inhibition with propranolol sensitized cancer cells to rapamycin. These data indicate that lipin-1 controls main cellular processes involved in cancer progression and that its targeting, alone or in combination with other treatments, could open new avenues in anticancer therapy.

## INTRODUCTION

Alterations of various metabolic pathways are frequently noticed in cancer cells. Among them, the most documented is increased glucose consumption through aerobic glycolysis known as the “Warburg effect”. However, other metabolic processes, such as protein, nucleic acid and lipid biosynthesis, are also deregulated in cancer cells [1]. This metabolic reprogramming is needed to meet the increased requirements of highly proliferating cancer cells for energy and building blocks. In the case of lipids, their increased biosynthetic rate provides material required for cell membranes formation and energy storage. In addition, lipids play also significant roles as signaling molecules. The alteration of their abundance can affect crucial processes necessary for cell transformation such as migration, invasion and tumor angiogenesis [2]. Finally, lipids are also required for protein modifications that critically regulate their functions and are involved in protein and organelle turnover through autophagy regulation [2]. Thus, the various roles of lipids make them essential components of the cellular machinery regulating the phenotype of cancer cells. Since the pivotal observation that Fatty Acid Synthase (FASN) is a potential target for anticancer therapy [3], much effort has been devoted to targeting key enzymes of lipid biosynthesis. Inhibition of fatty acid synthesis by pharmacological tools or targeting key enzymes with siRNA results in inhibition of cancer cell proliferation or cell death [4-7].

Although lipid homeostasis deregulation is observed in many different cancer types, it is especially critical in prostate cancer where the classical “glycolysis-switch” is not observed [8]. Targeting key enzymes of lipid biosynthesis appears therefore as a promising approach to fight prostate cancer [9]. However, numerous enzymes are involved in lipid biosynthesis and the specific role of many of them during cancer progression is still unknown [10]. This is the case for lipin-1, one of the three members of the lipins family. Lipin-1 is involved in the regulation of triglyceride and phospholipid biosynthesis by catalyzing the dephosphorylation of phosphatidate into diacylglycerol (DAG) [11]. It acts also as a co-regulator of transcription and, as such, can up-regulate fatty acids uptake and oxidation, TCA cycle and mitochondrial metabolism genes. Thus, due to its dual function as enzyme and co-transcriptional regulator, lipin-1 is able to regulate lipid homeostasis at several nodal points [12]. Very recently, it was also described as being involved in the late phase of autophagy [13], a key cellular function contributing to cancer progression in a context-dependent manner [14].

Here, we show that lipin-1expression is increased in various cancer cell types both *in vitro* and *in vivo* in human prostate tumor samples. The specific inhibition of lipin-1 in prostate and breast cancer cells demonstrates its critical importance for cell proliferation and migration through deregulation of several intracellular signaling pathways. This study demonstrates for the first time that the targeting of lipin-1 is a potential new anti-cancer strategy that could be used alone or in combination with drugs like rapamycin.

## RESULTS

### Expression of lipin-1 in cancer

**Figure 1:**
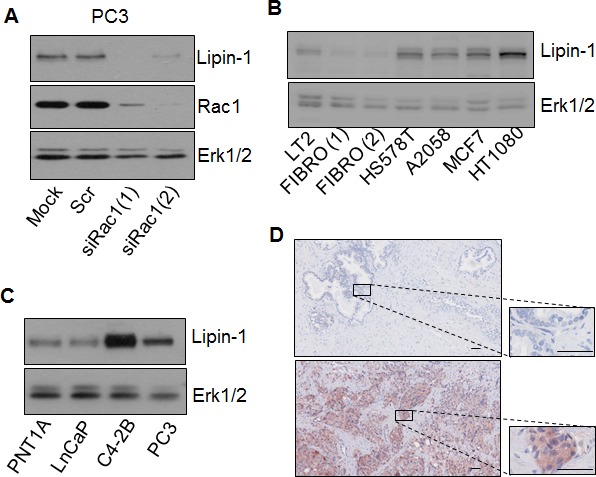
Lipin-1 expression is increased in various cancer cell lines and in prostate cancer samples (A) Lipin-1 is positively regulated by Rac1. 48 h after transfection with two different siRNA targeting Rac1 (siRac1(1) and siRac1(2)), with a control siRNA (Scr) or without sirna (mock) PC-3 cells were lysed and analysed by immuno-blotting with specific antibodies to lipin-1, Rac1 and Erk1/2. (B) Lipin-1 is highly expressed in various cancer cell lines as compared to fibroblasts and endothelial cells. Fibroblasts (FIBRO), endothelial cells (LT2), A2058, Hs578T, MCF7 and HT1080 cells were lysed and analysed by immuno-blotting with specific antibodies to lipin-1 and Erk1,2. (C) Lipin-1 is highly expressed in the most aggressive prostatic cancer cell lines. PNT1A, LnCaP, C4-2B and PC-3 cells were lysed and analyzed by immuno-blotting with specific antibodies to lipin-1 and Erk1,2. (D) Representative images of sections of normal human prostate (up) and of high grade prostate adenocarcinoma positive for anti-lipin-1 labelling (down) are shown. The 19 normal prostate tissues tested were negative while 16 out of 30 high-grade prostate adenocarcinomas were labelled with anti-lipin-1 antibodies. Bars = 50 μm.

We previously identified lipin-1 by microarray as a Rac1-regulated gene in the prostate adenocarcinoma cell line PC-3 (personal observation). This regulation was validated here at the protein level by silencing Rac1 with two different siRNA that resulted in lipin-1 down-regulation (Fig. 1A). RT-qPCR measurements indicate that Rac1 silencing significantly decreased (δδCt of -0.9) the lipin-1 gene expression confirming that this regulation occurred, at least partly, at the transcriptional level. As Rac1 is frequently over-expressed or over-activated in cancers [15-18], we reasoned that lipin-1 might also be over-expressed in various cancer cell lines as compared to normal skin fibroblasts or endothelial cells (Fig. 1B and 1C). Its expression was stronger in the highly tumorigenic PC-3 and C4-2B prostatic cell lines than in the low- or non-tumorigenic prostatic cells (LnCaP and PNT1A, respectively). Lipin-1 was also found highly expressed in prostatic cancers *in vivo* since 16 out of 30 high-grade human prostate adenocarcinomas were stained with anti-lipin-1 antibodies. By contrast, the 19 tested normal prostate tissues were all negative. As illustrated in Fig. 1D, the staining was observed only in epithelial cells and never in stromal cells and was almost exclusively cytoplasmic.

### Lipin-1 silencing repressed cell proliferation in cancer cells

**Figure 2:**
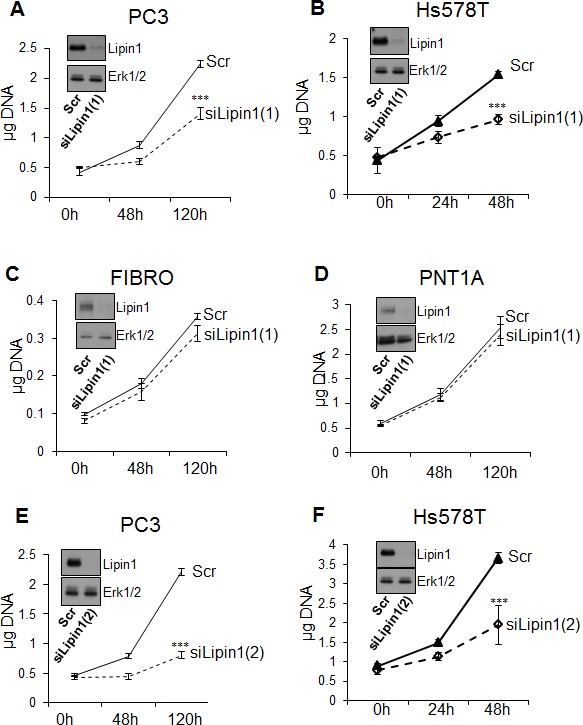
Lipin-1 silencing repressed proliferation of prostate adenocarcinoma and breast adenocarcinoma cells (PC-3 and Hs578T) but not proliferation of normal prostate epithelial cells (PNT1A) and human fibroblasts (FIBRO) Immediately after transfection with a control siRNA (Scr) or with a siRNA targeting lipin-1 (siLipin1(1) or siLipin1(2)) cells were seeded in 24-well plates and collected at the indicated times. The DNA content of each well was measured as described in “Materials and Methods”. ***: p< 0.001 as determined by ANOVA followed by Tukey-Kramer analysis.

Lipin-1 was silenced by RNAi to evaluate its importance for cell phenotype. As observed by Western blot analysis, lipin-1 expression was strongly repressed after transfection with specific siRNA in all cell types tested in the proliferation assay (Fig. 2). This inhibition lasted for at least 4 days and started to recover at day 5 post-transfection (Supplemental Fig. 1). Lipin-1 inhibition did not affect PC-3 cell survival, as assessed by apoptosis measurements (Supplemental Fig. 2), but repressed their proliferation rate as assessed by DNA measurements and cell counting (Fig. 2A and Supplemental Fig. 3). This effect was not limited to PC-3 cells as silencing of lipin-1 in breast adenocarcinoma cells (Hs578T) also reduced significantly their proliferation rate (Fig. 2B). Despite an efficient silencing of lipin-1, control cells (normal human skin fibroblasts and the non-tumorigenic prostatic cell line PNT1A) were not affected by lipin-1 repression suggesting that non-tumorigenic cells are less sensitive to lipin-1 depletion (Fig. 2C and Fig. 2D). The second lipin-1 siRNA that is more efficient in silencing lipin-1 tends also to be more efficient in reducing cancer cell proliferation (compare Fig. 2A to Fig. 2E and Fig. 2B to Fig. 2F). A similar repression of proliferation of PC-3 cells was also observed in lipid-free medium suggesting that extracellular lipids are not involved in this process (Supplemental Fig. 4).

### Lipin-1 silencing represses PC-3 cells migration through RhoA activation

**Figure 3:**
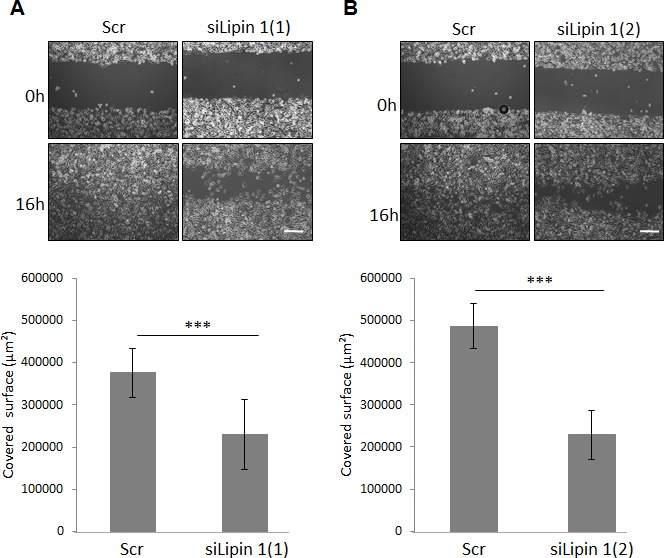
Lipin-1 silencing repressed cell migration Immediately after transfection with the indicated siRNA, cells were processed for the migration assay as described in “Materials and Methods”. Representative phase contrast micrographs were taken immediately after releasing the insert (0 h) and 16 hours later (16 h). Bar = 250 μm. ***: p<0.001 as determined by ANOVA followed by Tukey-Kramer analysis.

**Figure 4:**
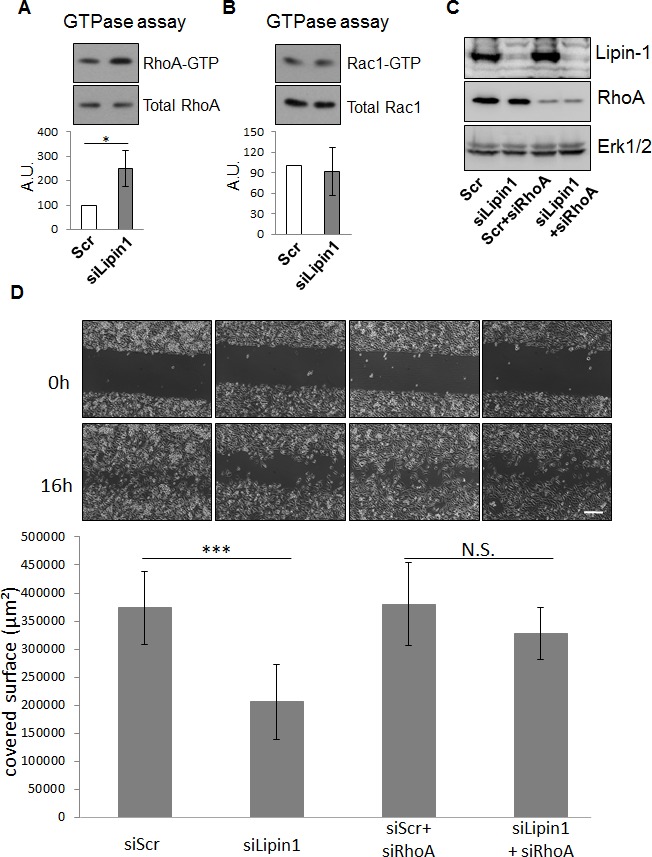
Lipin-1 silencing increased RhoA activity while Rac1 activity was not altered 48 h after transfection with the indicated siRNA, cells were processed for the GTPase activity assay as described in “Materials and Methods” and Western blot analysis with specific antibodies to RhoA, Rac1, and Erk1/2. Representative analyses for RhoA (A) and Rac1 (B) activity are illustrated. The results of each graph are expressed as mean ± s.d. of three independent experiments. (C-D) The repression of migration following Lipin-1 silencing is rescued by co-silencing of RhoA. Immediately after transfection with a control siRNA (Scr), a siRNA targeting lipin-1 (siLipin1), a control siRNA and a siRNA targeting RhoA (scr+siA1) or with a siRNA targeting lipin-1 and a siRNA targeting RhoA (siLipin1+siRhoA), cells were processed for the wound healing assay as described in “Materials and Methods”. An aliquot of the cell suspension was seeded in a dish and collected 48 h after transfection for western blot analysis with specific antibodies to lipin-1, RhoA and Erk1/2 to control the efficiency of silencing (C). (D) Representative phase contrast micrographs were taken immediately after releasing the insert (0 h) and 16 hours later (16 h). Bar = 250 μm. N.S.: not significant, *: p<0.01 and ***: p<0.001 as determined by ANOVA followed by Tukey-Kramer analysis. The graphs summarize the results of three independent experiments expressed as means ± s.d.

To further evaluate the relevance of the silencing of lipin-1 on cellular functions involved in tumorigenesis, we evaluated its effect on the migratory properties of PC-3 cells. In a scratch wound healing assay, lipin-1 silencing decreased the migration of PC-3 cells (Fig. 3). As RhoGTPases are key regulators of cellular migration [19], the level of the active form of RhoA and Rac1 was measured by mean of a pull-down assay. We observed that the activity of RhoA was increased following lipin-1 silencing (Fig. 4A) while the activity of Rac1 was not significantly altered (Fig. 4B). As we previously reported that an excess of RhoA activity can repress migration of PC-3 cells [20], a simultaneous repression of lipin-1 and RhoA was performed. As illustrated in Fig. 4C, both proteins were efficiently silenced and the repression of cell migration due to lipin-1 silencing was abolished (Fig. 4D). RhoA silencing alone did not affect the migration rate, indicating that the reversal of the inhibitory effect of lipin-1 silencing is not due to a nonspecific increase of the migration rate (Fig. 4D). By contrast, the depletion of RhoA did not reverse the effect of lipin-1 silencing on proliferation (Supplemental Fig. 5).

### Lipin-1 silencing regulates genes and pathways involved in cell metabolism

**Figure 5:**
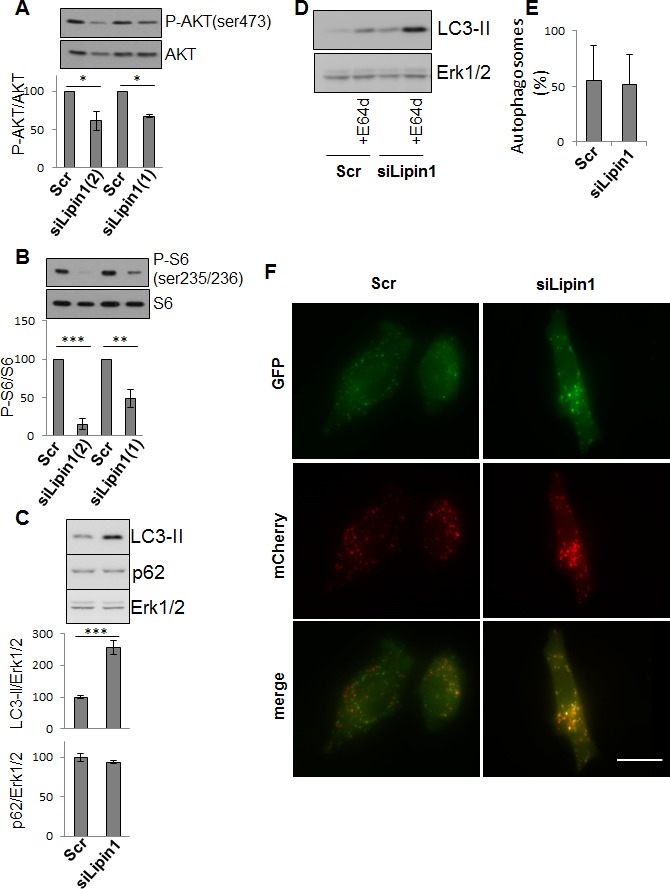
Lipin-1 silencing inhibited AKT and ribosomal protein S6 phosphorylation and enhanced autophagy PC-3 cells were transfected with the indicated siRNA. (A-D) 48 hours after transfection, cells were processed for Western blotting and analyzed with specific antibodies to AKT, Phospho-AKT (ser473), ribosomal protein S6, phospho ribosomal protein S6 (ser235/236), LC3 and Erk1/2. The results of each graph are expressed as mean ± s.d. of three independent experiments. In D, cells were treated or not with 10 μg/ml of the lysosomal protease inhibitor E64d (+E64d). In E and F, cells were first transfected with an expression vector for RFP-GFP-LC3B as described in “Materials and Methods” and after transfected with the indicated siRNA. In E, the graph represents the percentages of autophagosomes calculated in more than 50 cells per condition as described in “Materials and Methods” expressed as mean ± s.d. In F, representative fluorescent micrographs are shown. Bar = 10 μm *: p<0.05 and ***: p< 0.001 as determined by ANOVA followed by Tukey-Kramer analysis.

Lipin-1 has emerged as a crucial lipid regulator acting either as an enzyme or a co-regulator of transcription [12]. The silencing of lipin-1 did neither significantly affect the whole lipid synthesis nor triglyceride concentration nor the expression of fatty acid synthase but increased the cellular concentration of phosphatidic acid (PA) (Supplemental Fig. 6). By contrast, it repressed the mRNA expression of ATP citrate lyase (Supplemental Fig. 6), a cytosolic enzyme that catalyzes the generation of acetyl-CoA from citrate and is over-expressed in several types of cancers [6]. A decreased phosphorylation of AKT and of ribosomal protein S6 was also observed upon lipin-1 silencing (Fig. 5A and Fig. 5B). Autophagy is often induced in cancer cells in order to maintain a high metabolic rate [14]. We observed that the repression of lipin-1 induced an accumulation of LC3-II while p62/SQSTM1 level was not altered (Fig. 5C) and the amount of LC3-II further increased in the presence of the lysosomal inhibitor E64d (Fig. 5D). Moreover, cells expressing a double tagged LC3 (RFP-GFP-LC3) and silenced or not for lipin-1 displayed comparable percentages of autophagosomes (Fig. 5E and Fig. 5F), suggesting that the accumulation of LC3-II is not due to a blockage of autophagosome maturation. These data support an enhanced autophagic flux following lipin-1 silencing that could help cells to counteract the negative effect of lipin-1 down-regulation.

### Manipulation of lipin-1 amount induced a compensatory regulation of lipin-2

**Figure 6:**
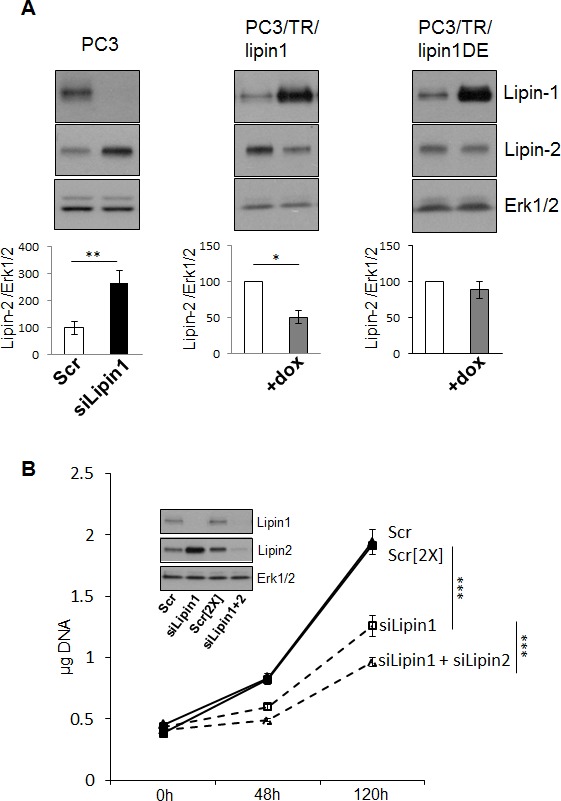
Compensatory regulation between lipin-1 and -2 affects PC-3 phenotype (A) There is a compensatory regulation of lipin-2 protein level following modulation of lipin-1 protein level that is dependent on lipin-1 activity. 48 hours after transfection of the indicated siRNA in PC-3 cells or after induction (+dox) of the expression of lipin-1 (in PC-3/TR/lipin1) or of the expression of inactive lipin-1 (in PC-3/TR/lipin-1DE), cells were lysed and analyzsed results of three independent experiments. (B) The inhibition of proliferation mediated by lipin-1 silencing was enhanced following co-silencing of lipin-2. Immediately after transfection with 20 (Scr) or 40 (Scr[2X]) nM of a control siRNA, 20 nM of the first siRNA targeting lipin-1 (siLipin1) or 20 nM of the first siRNA targeting lipin-1+ 20 nM of an siRNA targeting lipin-2 (siLipin1+siLipin2) cells were seeded in 24-well plates and collected at the indicated times. The DNA content of each well was measured as described in “Materials and Methods”. The insert shows Western blot analysis of cell lysates collected 48 h after transfection with the indicated siRNA with specific antibodies to lipin-1, lipin-2 and Erk1/2. *: p<0.05, **: p<0.01 and ***: p< 0.001 as determined by ANOVA followed by Tukey-Kramer analysis.

A reciprocal regulation between lipin-1 and -2, but not lipin-3, was recently reported [21]. As lipin-1 and lipin-2 share common functions, an increased lipin-2 expression could partly rescue lipin-1 silencing. As illustrated in Fig. 6A, silencing of lipin-1 induced an increased expression of lipin-2 in PC-3 cells. This regulation was also observed in Hs578T cells but not in human fibroblasts or in control prostatic PNT1A cells (Supplemental Fig. 7). On the reverse, over-expression of lipin-1 by clones expressing lipin-1 (PC-3/TR/Lipin1) in a doxycycline-dependent way induced a compensatory down-regulation of lipin-2. This effect depends on the catalytic activity of lipin-1 since over-expression of the phosphatase-dead mutant lipin-1D678E does not influence the level of lipin-2. The low level of lipin-3 expression relative to that of lipin-1 and lipin-2 (~10 fold lower) as evaluated by RT-qPCR and the lack of compensatory mechanism with the two other lipins [21] motivated us to not further investigate lipin-3 here.

### Lipin-1 and lipin-2 cooperate to regulate cell proliferation but not cell migration

In order to investigate the consequences of the compensatory increased level of lipin-2 following lipin-1 silencing, we targeted also lipin-2 through RNAi (insert, Fig. 6B). The double silencing of lipin-1 and lipin-2 was very efficient and inhibited cell proliferation more strongly than the lipin-1 silencing alone, which shows that lipin-2 can partially compensate for the lack of lipin-1 in this model. By contrast, the inhibition of migration of PC-3 cells was not affected by the depletion of lipin-2 (Supplemental Fig. 8). Altogether, these data demonstrate that lipin-1 and lipin-2 are both involved in the control of tumoral cells proliferation but that only lipin-1 regulates cell migration.

### Pharmacological inhibition confirmed the role of lipins in the regulation of cell phenotype

**Figure 7:**
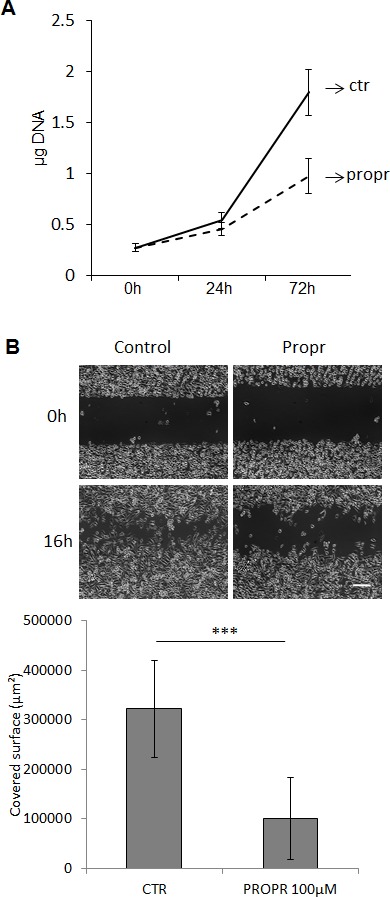
Propranolol inhibited PC-3 cell proliferation and migration PC-3 cells were treated with 100 μM propranolol (propr). (A) The DNA content of each well was measured as described in “Materials and Methods”. (B) Cells were processed for the migration assay as described in “Materials and Methods”. Propranolol was added just after the removal of the inserts. Representative phase contrast micrographs were taken immediately after releasing the insert (0 h) and 16 hours later (16 h). Bar = 250 μm. ***: p<0.001 as determined by ANOVA followed by Tukey-Kramer analysis.

**Figure 8:**
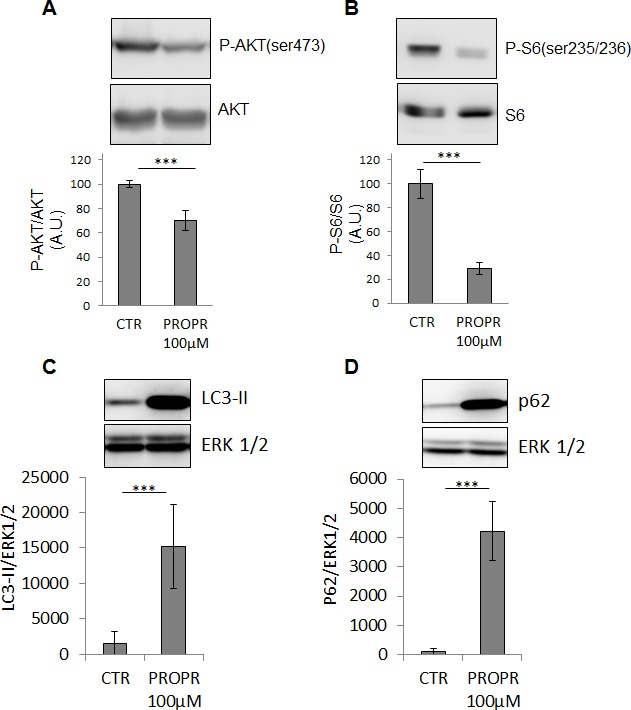
Propranolol inhibited AKT and S6 phosphorylation and induced LC3II and p62 accumulation 24 h after treatment with 100 μM propranolol, PC-3 cells were processed for Western blotting and analysed with specific antibodies to AKT, Phospho-AKT (ser473), ribosomal protein S6, phospho-ribosomal protein S6(ser235/236), LC3, p62/SQSTM1 and Erk1/2. The results of each graph are expressed as means ± s.d. of three independent experiments. ***: p< 0.001 as determined by ANOVA followed by Tukey-Kramer analysis.

As a chemical alternative to RNAi, lipins activity can also be inhibited by propranolol [22-24]. Pharmacological concentrations of propranolol significantly inhibited PC-3 cell proliferation and migration (Fig. 7A and Fig. 7B). Propranolol also repressed AKT and S6 protein phosphorylation (Fig. 8A and Fig. 8B). An accumulation of LC3-II was also noted upon propranolol treatment but, on the contrary to lipin-1 silencing, it induced an accumulation of p62 suggesting a blockage in the late phases of autophagy (Fig. 8C and Fig. 8D). This discrepancy with lipin-1 silencing alone is most likely due to the fact that propranolol completely inhibits all lipins while specific lipin-1 depletion with siRNA causes induction of lipin-2. As a whole, pharmacological inhibition of lipins confirmed their role in the regulation of the cell phenotype.

### Lipin-1 depletion or inhibition sensitizes cancer cells to rapamycin treatment

**Figure 9:**
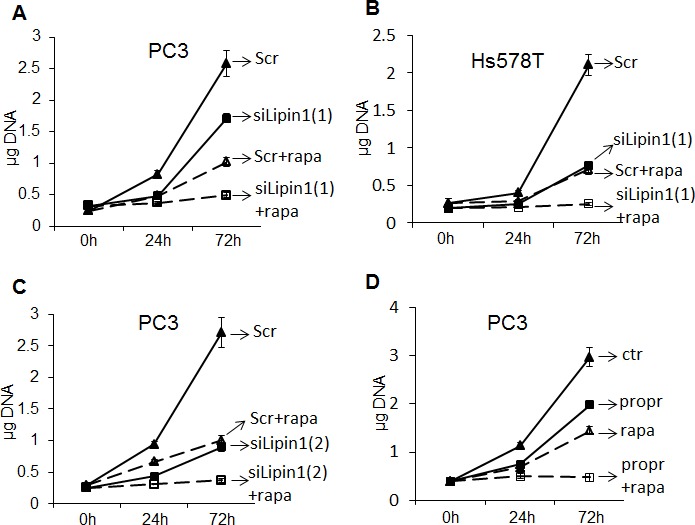
Potentiation of the anti-proliferative effect of rapamycin by depletion or pharmacological inhibition of lipin-1 (A-C) Immediately after transfection of prostate adenocarcinoma (PC-3) or breast adenocarcinoma cells (Hs578T) with a control siRNA (Scr) or with an siRNA targeting lipin-1 (siLipin1(1) or siLipin1(2)) cells were seeded in 24-well plates and collected at the indicated times. Where indicated, the cells were cultured with 50 nM of rapamycin (+rapa). In D, PC-3 cells were treated with 50 nM rapamycin (rapa) and/or 100 μM propranolol (propr). The DNA content of each well was measured as described in “Materials and Methods”. *** p< 0.001 ANOVA followed by Tukey-Kramer analysis.

Rapamycin is a widely used anti-cancer agent. However, when used alone, it has often limited effects mainly due to the loss of negative feedback loops in cancer cells leading to reactivation of AKT and ribosomal protein S6. As we observed that phosphorylation of AKT and ribosomal protein S6 were decreased following lipin-1 silencing, we hypothesized that lipin-1 silencing could potentiate the anti-proliferative effect of rapamycin on cancer cells. A preliminary experiment showed that rapamycin treatment of PC-3 cells in a range of 10 nM to 1 μM induced a similar repression of proliferation. In the following experiments, concentrations of 20 and 50 nM of rapamycin were used and gave similar results. Only the data with the 50 nM concentration are shown. While rapamycin treatment of PC-3 (Fig. 9A and Fig. 9C) and Hs578T cells (Fig. 9B) reduced their proliferation rate by about 60 %, the combination of rapamycin treatment with lipin-1 silencing almost completely suppressed their proliferative capacities. Pharmacological concentrations of propranolol, alone or in association with rapamycin, also repressed cell proliferation by 40 or almost 100 %, respectively (Fig. 9D) which further demonstrated the synergistic effect of targeting these two pathways. The potentiation of the effect of rapamycin by propranolol was confirmed by direct cell counting (Supplemental Fig. 9).

## DISCUSSION

Deregulation of lipid metabolism in various cancer types makes it an attractive target for anti-cancer therapies. A first strategy to limit fatty acid availability would consist in inhibiting lipid synthesis [10]. This approach was largely used in recent research and led to the identification of several key enzymes for which targeting affected cancer cell proliferation and survival. Based on these data, specific inhibitors have been developed and are currently being tested in clinic (reviewed by [4-7]). Fatty acids level can also be decreased by enhancing their degradation, increasing their storage or blocking their release from storage. In the lipid storage pathway, lipin-1 is one of the least studied enzymes in cancerogenesis [10]. We identified lipin-1 as a Rac1 regulated gene, probably through mTORC1 activation [25] that, in turn, regulates positively SREBP1 [26], a transcription factor involved in lipin-1 gene expression [27]. Via mTORC1, Rac1 could also regulate lipin-1 phosphorylation and, consequently, its activity and its localization [28]. The high expression of lipin-1 in various cancer cell lines as well as its over-expression noticed in about 50 % of high grade prostate cancer samples prompted us to characterize its role in cancer cell phenotype. Most of our study was performed with prostate cancer cells as this type of cancer is especially dependent on an enhanced lipid metabolism [29]. In addition, lipin-1 depletion strongly inhibited cancer cells proliferation while it had only limited effect on non-cancerous cells. This is a key point for the clinical relevance of the targeting of this protein. The inhibition of lipin-1 did not significantly affect whole lipid synthesis and FASN expression. Lipin-1 is expected to regulate FASN expression through the inhibition of SREBP1 activity. However, in our model, this transcriptional function of lipin-1 is likely repressed as lipin-1 seems highly phosphorylated by a mTOR-dependent pathway (Supplemental Fig. 10A), a modification reported to prevent its entry into the nucleus [30]. This is in line with our observations showing that lipin-1 is exclusively located in the cytoplasm and never in the nucleus of high-grade adenocarcinoma cells in tumor samples. In prostate cancer cells, lipin-1 silencing is correlated with PA accumulation and reduced proliferation, while many reports indicate that increased concentration of PA is often correlated with a proliferative phenotype (for review see [31]). However, these data are not contradictory when considering the mechanisms leading to the increased intracellular levels of PA. When high concentration results from the stimulation of its synthesis (via phospholipase D, lysophosphatidic acid acetyltransferase or diacylglycerol kinase), it induces an increased proliferation rate. In our model, accumulation is due to a decrease in its conversion into DAG, which affects both PA- and DAG-dependent signaling pathways and further impairs the production of phospholipids (phosphatidylethanolamine, phosphatidylcholine, …) used as building blocks for cell membranes. This mechanism has been proposed to explain the induction of cell death in U937 cells by a semi-specific inhibitor targeting lipin-1 [32]. It has been shown that lipin-1 is induced in culture conditions where PA is up-regulated such as serum withdrawal (Supplemental Fig. 10B and^33^]) or hypoxia [34, 35] but also in response to exogenous PA (Supplemental Fig. 10C) likely to regulate lipid homeostasis. Thus, lipin-1 depletion is likely to trigger both an imbalance between lipid species and a decrease in nutrient availability that might explain the decreased phosphorylation of AKT and ribosomal protein S6 and the increase in autophagy required to fuel the cells with building blocks and energy. Importantly, the loss of lipin-1 was recently reported to inhibit the clearance of autophagy in muscle cells [13]. However, this process does not seem to operate in prostate cancer cells depleted in lipin-1. The enhanced level of LC3-II noticed upon lipin-1 silencing was not associated with an up-regulation of p62/SQSTM1. In addition, we did not observe any accumulation of autophagosomes at the expense of autolysosomes. This discrepancy is likely related to our experimental model in which lipin-1 depletion is partially compensated by lipin-2 over-expression. This hypothesis is further supported by the observation that propranolol, a pharmacological molecule that completely inhibits all lipins, also induced both LC3II and p62/SQSTM1 accumulation in PC-3 cells.

The inhibition of cell migration following lipin-1 silencing further enhanced the relevance of lipin-1 to tumorigenesis. Here, we have demonstrated that this effect is related to an increased activation of RhoA, a data in agreement with previous report from our research group and others demonstrating that an increased activation of RhoA is sufficient to repress PC-3 cell migration [20, 36]. This RhoA activation in lipin-1-depleted cells could be related to the increased concentration of PA which can activate the sphingosine kinase-1 to produce sphingosine-1-phosphate [37], a lipid mediator able to enhance RhoA activity [38], and to inhibit migration of various tumor cells including PC-3 [36, 38].

It was recently reported that lipin-1 depletion results in a reciprocal increase in lipin-2 but not lipin-3 expression [21]. A similar regulation was also observed here in prostate cancer cells suggesting that this mechanism is widespread. We further showed that overexpression of lipin-1 represses lipin-2 expression and that this feedback dynamic compensation is dependent on the catalytic activity of lipin-1, a mechanism that has to be taken into account in any strategy aiming at targeting lipins.

The PI3K/AKT/mTOR pathway is over-activated in several cancers including prostate cancers [39] and much effort has been devoted to its inhibition. Inhibition of the mTOR complex 1 with rapamycin or its analogs exerts an anti-tumoral activity in tumor cell lines and therapeutic response has been reported for some cancers [40-42]. However, in numerous cancer types including prostate cancer, mTOR complex 1 inhibition by rapamycin had a limited therapeutic efficacy in part due to the loss of negative feedback loops leading to the activation of AKT by TORC2 [43]. To overcome this resistance, rapamycin has been successfully combined with other drugs such as aromatase inhibitors [44] or HDAC inhibitors [45-48]. Thus, the identification of proteins whose silencing synergizes with mTOR inhibition to decrease tumor growth is of particular interest [49]. The inhibition of AKT and ribosomal protein S6 activation following lipin-1 silencing was the rationale to associate lipin-1 siRNA and rapamycin treatment. Since our data demonstrated that lipin-1 silencing sensitizes prostate and breast cancer cells to rapamycin, we then verified whether pharmacological inhibition of lipins activity could be considered as a potential therapeutic option or not. Propranolol was initially used as a beta-adrenoceptor antagonist. It displays anti-tumor activity in neuroblastoma [50] and can potentiate chemotherapy in breast cancer [51]. It was later on found to be a potent inhibitor of lipins activity. Used alone, it recapitulates most of the effects of lipin-1 silencing on PC-3 cells phenotype. Most interestingly, it fully sensitizes PC-3 cells to rapamycin treatment, similarly to what was observed by using a siRNA targeting lipin-1. Thus, our data opens the way to new anti-cancer strategies, especially by providing the rationale to combine two well-known molecules in human therapy, propranolol and rapamycin, for the treatment of cancer.

## MATERIALS AND METHODS

### Reagents and cells

Bisbenzimide H 33258 was from Calbiochem (Merck, Overijse, Belgium). 1,2-Dioctanoyl-*sn*-glycerol 3-phosphate sodium salt (P3591), rapamycin (R8781) and (+)-propranolol (P0884) were from Sigma. Rabbit anti-lipin-1 (#sc-98450), rabbit anti-lipin-2 (#sc-134433), mouse anti-ribosomal protein S6 (#sc74459), mouse anti-phospho ribosomal protein S6 (ser235/236) (#sc293144), rabbit anti-p62/SQSTM1 (#sc28359) and mouse anti-RhoA (#sc-418) were from Santa-Cruz Biotechnology (Bioconnect, The Netherlands). Mouse anti-Rac1 (23A8) was from Upsate Biotechnology. Mouse anti-human LC3 (#0260-100) was from Nanotools (Merck, Overijse, Belgium). Rabbit anti-AKT (#9272) and rabbit anti-phosphoAKT(ser473) (#9271) were from Cell Signalling (Bioke, The Netherlands). Rabbit anti-Erk1/2 (#M-5670) and mouse anti-phosphoErk1/2 (#M8159) were from Sigma. The secondary horseradish peroxydase conjugated rabbit anti-mouse IgG (P0260) and swine anti-rabbit IgG (P0217) were from DAKO (Heverlee, Belgium). Human prostate adenocarcinoma cells PC-3 were cultured in F-12 Kaighn's medium (Invitrogen, Merelbeke, Belgium) supplemented with 7% Foetal Bovine Serum (FBS) (Lonza, Verviers, Belgium). Human prostate carcinoma cells (LnCaP) and human immortalized prostatic cells (PNT1A) were cultured in RPMI 1640 (Lonza) supplemented with 7% FBS. Human breast adenocarcinoma cells (Hs578T and MCF7), fibrosarcoma cells (HT1080), melanoma cells A2058, skin fibroblasts (FIBRO) and endothelial cells (LT2; human umbilical vein endothelium cells (HUVEC) immortalized by transfection with Large T SV40 antigen (a kind gift of E. Dejana, Milan)) were cultured in DMEM (Lonza) supplemented with 7% FBS. PC-3, Hs578T and MCF7 cells were authenticated through DNA profiling of 8 different and highly polymorphic short-tandem repeat loci (DSMZ, Braunschweig, Germany) in November 2013.

### siRNA transfection

21-nucleotides long siRNAs chemically synthesized, desalted, deprotected and PAGE purified were from Eurogentec (Liège, Belgium). The sequences of the siRNAs targeting RhoA (siRhoA), Rac1 (siRac1 and siRac1#2) and of the control siRNA (siScr) were described and validated previously [20, 52]. The sequences of siRNA used for repressing lipin-1 or lipin-2 expression were the followings: siLipin1#1 (5′-GAAUGGAAUGCCAGCUGAATT-3′ and 5′-UUCAGCUGGCAUUCCAUUCTT-3′), siLipin-1#2 (5′-GAGAGAUGACAACAUGAACTT-3′ and 5′-GUUCAUGUUGUCAUCUCUCTT-3′) and siLipin-2 (5′-GAUGGCAGCUAUCAGUGUUTT-3′ and 5′-AACACUGAUAGCUGCCAUCTT-3′). Each pair of oligoribonucleotides was annealed at a concentration of 20 μM in 50 mM NaCl, 1mM EDTA, 10 mM Tris-HCl pH 7.5. siRNA transfection was carried out as previously described [53]. Briefly, calcium phosphate-mediated transfection was performed overnight (14-16 h) on subconfluent cells at a final concentration of 20 nM siRNA. Cells were washed twice with PBS and once with complete medium, this last step being defined as time 0 post-transfection. Cells were lysed for Western blot or RT-qPCR analysis 48 hours post-transfection.

### Western blotting

Cells were lysed in SDS-PAGE lysis buffer and proteins were separated by polyacrylamide gel electrophoresis. Proteins were transferred to a PVDF Transfer Membrane (NEN Life Science Products). Membranes were then blocked for 1 hour with 3 % dry milk in PBS-0.05 % Tween 20 and incubated for 4 h with the diluted primary antibody. Membranes were then washed three times, incubated in the diluted secondary horseradish peroxydase-conjugated antibody for 1 h, and revealed by chemoluminescence using the ECL kit (Amersham Biosciences) and X-ray film exposure. The membranes were re-probed with anti-Erk1/2 antibodies to control protein loading.

### Immunohistochemistry

This protocol was established based on manufacturer's instructions. Human prostate carcinoma paraffin embedded sections (5 μm) were dewaxed and rehydrated using the following successive baths: 8 min in xylol, 4 min in xylol, 2 min in 100 % ethanol, 2 min in 95 % ethanol, 2 min in 70 % ethanol and 2 min in H_2_O. Antigen retrieval was performed by heating samples in REAL^tm^ Retrieval Solution (Dako) at 120°C for 5 min. After cooling, endogenous peroxydases were inhibited using 3 % H_2_O_2_ during 20 min at room temperature (RT). Background staining was reduced by incubating the slides in 10 % FBS/PBS for 30 min at RT. Sections were then subsequently incubated with the primary anti-lipin-1 antibody (dilution 1/100) for 1 h at RT then with the biotin conjugated anti-rabbit antibody (dilution 1/400) during 45 min and then with HRP conjugated streptavidin (dilution 1/400) during 30 min at RT. Staining was revealed using AEC+ High Sensitivity Chromogen (Dako) and sections were counterstained with haematoxylin.

### Lipid synthesis, triglycerides and phosphatidic acid concentration measurements

Rates of lipid synthesis and fatty acid beta-oxidation were assessed in PC-3 cells 24 h after transfection with the indicated siRNA. The lipid assay was based on the method of Lin and collaborators [54] with some modifications. Briefly, 24 h after transfection, PC-3 cells were incubated with [^3^H]acetate (2 μCi/ml; Perkin Elmer; used as a metabolic precursor of lipids) during 24 h Lipids were extracted using the chloroform–methanol method, and lipid radioactivity was measured by liquid scintillation (Lipoluma; Lumac). Triglycerides and phosphatidic acid content were measured in cells 48 h after transfection with the indicated siRNA. Lipids were extracted using the chloroform-methanol method. Triglycerides were measured by using a a colorimetric assay (Sigma, TR0100) and the phosphatidic acid content by using a fluorometric assay kit (Cayman chemical, 700240) following the instructions of the manufacturer.

### Proliferation assay

Cells were seeded in 24 wells costar plates immediately after transfection with siRNA and collected at different time points to determine the DNA content by a fluorimetric technique [55] as previously described [56]. Briefly, cell layers were rinsed three times with saline and homogenized in PBS by sonication (20 s/well). 100 μl of each sample were transferred into a 96 well plate and supplemented with 100 μl of bisbenzimide solution (200 μg/ml bisbenzimide, 4 M NaCl, 20 mM NaH_2_PO_4_, pH; 7.4). In each plate a standard curve of DNA (from 2 μg to .03 μg) was included. The plate was mixed 5 min and fluorescence was read in a microplate spectrofluorometer SpectraMax Gemini XS (Molecular Devices, Sunnyvale, CA, USA) with an excitation wavelength of 355 nm and an emission wavelength of 460 nm.

For cell counting, cells were seeded in 12 wells costar plates immediately after transfection with siRNA. Cells were detached at different time points with 2.5 % trypsin-EDTA, pelleted and suspended in 300 μL of PBS. Flow cytometry was performed on a BD FACSVerse (405 nm, 488 nm, 633 nm, walk-away system) equipped with a “Optional Volumetric Flow Sensor” able to measure the volume over the entire acquisition time.

### Migration assay

PC-3 cells were seeded (3×10^4^ cells per well) into sterile Culture-Insert (#80209, ibidi). After 24 h the inserts were removed revealing a gap of 500 μm width in the cell monolayer. This was defined as the 0 h time of the assay. Phase-contrast micrographs of the gap were taken, at two random positions for each assay, at time 0 h and after 16 h using Nikon TiS microscope with a Clara High Resolution CCD camera (Andor), halogen Fiber Illuminator Intensilight, CFI Plan Fluor DL 10X 0.30 objective (Nikon) and controlled by NIS-Elements software (Nikon). The surface of the remaining gaps was measured using NIS-Elements software (Nikon) and the covered surface was calculated by subtracting the 16 h gap from the 0 h gap. Bar = 250 μm.

### GTPase activity assay

The assay was carried out as previously described [53, 57]. Briefly, cells were chilled on ice and lysed in ice-cold buffer containing 1 % Triton X-100, 25 mM HEPES pH; 7.3, 150 mM NaCl, 4 % glycerol, 0.1 mM AEBSF, 4 μg/ml aprotinin. Lysates were centrifuged for 6 min at 16000 g. Supernatants were immediately frozen in liquid nitrogen and stored at –80°C until used. An aliquot of each supernatant collected before freezing was denatured in SDS-PAGE lysis buffer to measure the total RhoGTPase content by Western blotting. For pull-down assays, supernatants were incubated for 30 min with 30 μg of GST-PBD protein containing the Cdc42 and Rac binding region of PAK-1B or 30 μg of GST-RBD protein containing the RhoA binding region of Rhotekin both affinity linked to glutathione-Sepharose beads. The beads were washed 4 times in lysis buffer and boiled in 60 μl SDS-PAGE lysis buffer.

### Real time quantitative PCR

Total RNA was isolated from siRNA-transfected cells using the High Pure RNA isolation kit (Roche Molecular Biochemical). 1 μg of total RNA was reversed transcribed using SuperScript III Reverse Transcriptase (Invitrogen). Real time qPCR was performed in a final volume of 20 μl containing 2 μl of cDNA (corresponding to 10 ng of total RNA for Lipin-1, Lipin-2, Lipin-3, FASN, ACLY and MAGL amplification and corresponding to 0.1 ng of total RNA for GAPDH amplification), 300 nM of each primer and 10 μl of the qPCR MasterMix Plus for SYBR^®^ green (Eurogentec) in the StepOne^TM^ Real-Time PCR system (Applied Biosystems, Halle, Belgium). The results were analyzed with the StepOne^TM^ Software and normalized to the GAPDH transcript.

### Generation of PC-3 clones overexpressing Lipin-1 or Lipin-1D678E under the dependence of doxycycline

The entire coding sequence of human Lipin-1 was amplified by RT-PCR (forward oligonucleotide 5′-cacacagaattcgcgccgctcggtgcagacca-3′, reverse oligonucleotide 5′-cacacactcgagtggcaagaggctgcttgggaca-3′) and cloned into the EcoRI/XhoI sites of pcDNA4/TO (Invitrogen) (pcDNA4/TO/Lipin1) and sequenced. The mutant Lipin1D678E was generated using a two-step PCR and two anti-complementary oligonucleotides possessing a point mutation (underlined in the sequences) as compared to the wild type sequence. Two overlapping fragments covering the entire coding sequence were first amplified from the pcDNA4/TO/Lipin1 using the following pairs of primers: 5′-cacacagaattcgcgccgctcggtgcagacca-3′ and 5′- gtcccatcaatctcagaaatgatgactttatcatccca-3′ (for the 5′PCR product) and 5′-cacacactcgagtggcaagaggctgcttgggaca-3′ and 5′- CATCATTTCTGAGATTGATGGGACAATTAC CAGATCA -3′ (for the3′PCR product). These two fragments were then mixed in equal amount, denatured, annealed and elongated before PCR amplification (using 5′-cacacagaattcgcgccgctcggtgcagacca-3′ and 5′-cacacactcgagtggcaagaggctgcttgggaca-3′ as forward and reverse primers). The final full size and mutated product was then digested, cloned into the pcDNA4/TO (pcDNA4/TO/Lipin1D678E) and sequenced to verify the presence of the mutation. Clones of PC-3 cells expressing a high level of tetracycline repressor (PC-3/TR) were previously described [52]. They were transfected with pcDNA4/TO/Lipin1 or pcDNA4/TO/Lipin1D678E and selected in medium supplemented with 1 μg/ml blasticidin + 200 μg/ml zeocin^TM^. Several clones overexpressing lipin-1 or lipin-1D678E in a doxycycline-dependent way (PC-3/TR/Lipin1, PC-3/TR/Lipin1D678E) were isolated and used in this study.

### Autophagy analyses

Autophagy flux was analyzed with the pBABE-puro mCherry-EGFP-LC3B reporter plasmid purchased from Qiagen and previously described [58]. PC-3 cells were seeded in a 6 well plate. 24 hours after seeding cells were transfected with 1μg of plasmid for 20-24 h with 3 μL GeneJuice^TM^ (Novagen) following the manufacturer's protocol. The cells were washed and directly transfected with the indicated siRNA as described above. After 16 h the cells were washed again and seeded at subconfluence in μ-Slides 8-well (Ibidi). Images were acquired by fluorescence microscopy and the analyses were performed using ImageJ software. Micrographs of the cells were acquired in several focal planes and the analysis performed on the stacked images. Puncta structures mCherry-positive and expressing or not EGFP were quantified in more than 50 cells per condition. The proportion of autophagosomes was expressed as the percent of puncta with both colors.

### Cell survival and apoptosis

Cell survival and apoptosis were evaluated by fluorescence-activated cell sorting after annexin V–FITC and Propidium Iodide staining. Adherent cells were detached with 2.5 % trypsin-EDTA, pelleted and suspended in Annexin binding buffer (Annexin V-FITC Apoptosis Detection kit, Sigma) and incubated for 15 min with Annexin V-FITC (270 ng/ml) and Propidium Iodide (1.1 μg/ml). Flow cytometry was performed on a FACSCanto II double LASER flow cytometer (UV, 488 nm, 633 nm) (BD Biosciences) and data were analyzed using FACSDiva Software (BD Biosciences).

## SUPPLEMENTARY MATERIALS, FIGURES


