# Supplementary material for: Lipin-1 regulates cancer cell phenotype and is a potential target to potentiate rapamycin treatment
Source: Oncotarget. 2015 Mar 14;6(13):11264–80. doi: 10.18632/oncotarget.3595 (PMC4484455; doi:10.18632/oncotarget.3595)
Supplement: Supplementary file 1 [file oncotarget-06-11264-s001.pdf]

# Lipin-1 regulates cancer cell phenotype and is a potential target to potentiate rapamycin treatment

## Supplementary Material

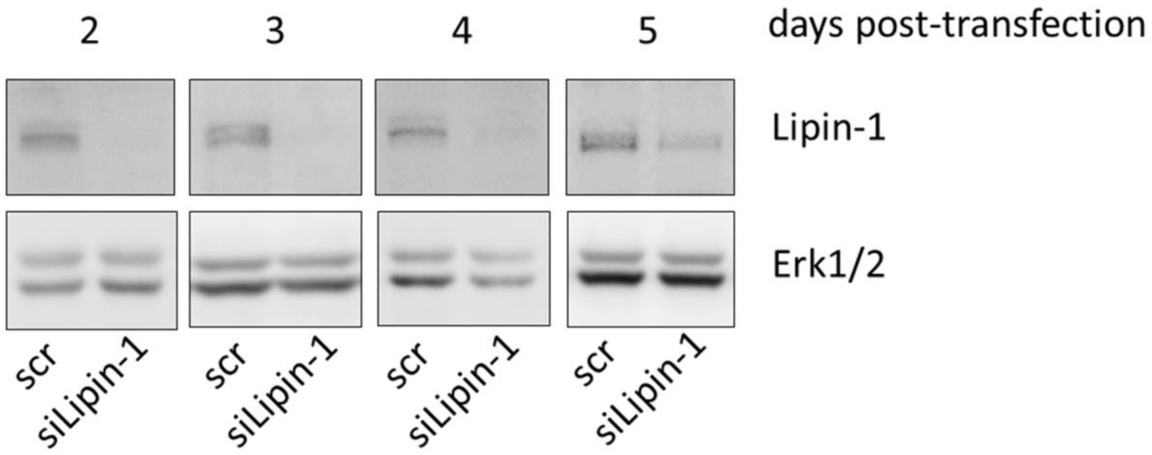


Supplemental Fig. 1: Time-course of lipin-1 silencing. Western blot analysis of whole-cell lysates of PC-3 cells transfected with the indicated siRNA. Cells were processed between day 2 and 5 after transfection and analyzed by immunoblotting with specific antibodies to lipin-1 and Erk1/2. Representative blots are shown.

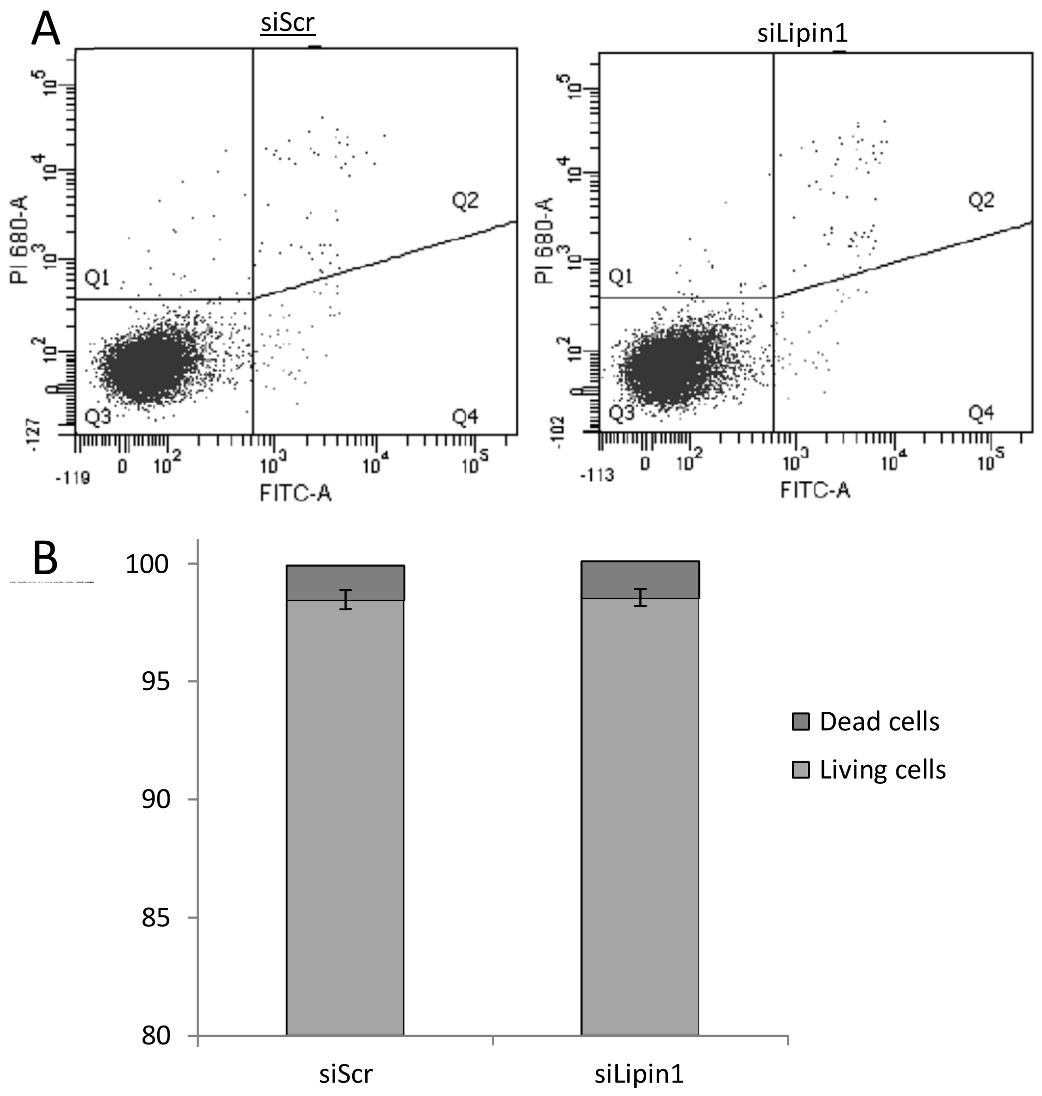


Supplemental Fig.2: FACS analysis was performed after labeling PC-3 cells with FITC-annexin V (FITC-A, X-axes) and propidium iodide (PI, Y-axes). 10.000 events were collected for each experiment. (A) Example of dots graphs of control cells (siScr) and cells depleted in lipin-1 (siLipin1). Alive cells [Q3] (double negative staining), cells in early apoptosis [Q4] (annexin V positive, PI negative), in late apoptosis [Q2] (double positive) and necrotic [Q1] (annexin V negative, PI positive) are indicated on the graphs. (B) Percentages of living / dead cells measured by FACS analysis. The dead cells include necrotic cells + early and late apoptotic cells. The graph summarizes the results of three independent experiments expressed as the mean±s.d.

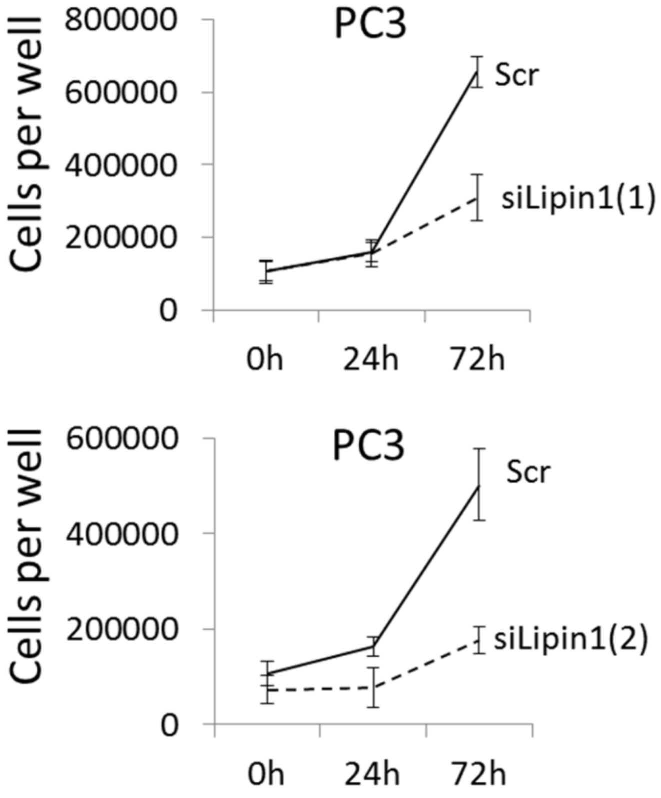


Supplemental Fig. 3: Lipin-1 silencing inhibits PC-3 cells proliferation as determined by cell counting. Immediately after transfection with a control siRNA (Scr) or with a siRNA targeting lipin-1 (siLipin1(1) or siLipin1(2)) cells were seeded in 12-well plates and collected at the indicated times after trypsin-EDTA treatment. The number of cells in each well was measured as described in “Materials and Methods”. ***: $p < 0.001$ as determined by ANOVA followed by Tukey-Kramer analysis.

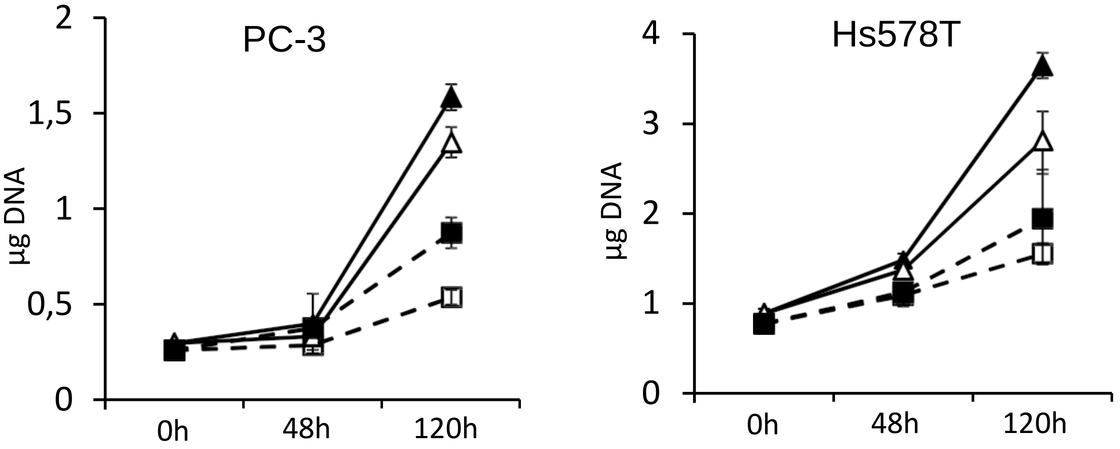


Supplemental Fig.4: The inhibition of proliferation following lipin-1 silencing is observed in normal and in lipid-free medium. Cells (PC-3, prostate adenocarcinoma; Hs578T, breast adenocarcinoma) were transfected with control (triangles) or lipin-1 specific (squares) siRNA and cultured in normal medium (black symbols) or in medium supplemented with lipid-free serum (open symbols). The DNA content of each well was measured as described in "Materials and Methods". *** p< 0.001 ANOVA followed by Tukey-Kramer analysis.

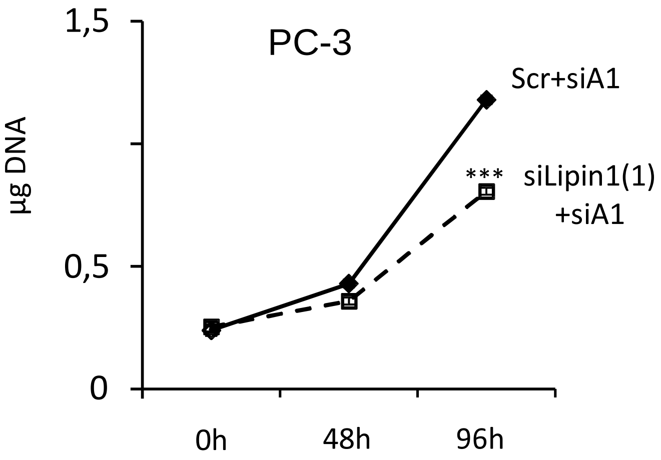


Supplemental Fig.5. The inhibition of proliferation following lipin-1 silencing is not rescued by RhoA silencing. Immediately after transfection with control siRNA + RhoA siRNA (Scr+siA1) or with lipin-1 siRNA + RhoA siRNA (siLipin1+siA1), cells were seeded in 24-wells plates and collected at the indicated times. The DNA content of each well was measured as described in "Materials and Methods". *** p< 0.001 ANOVA followed by Tukey-Kramer analysis.

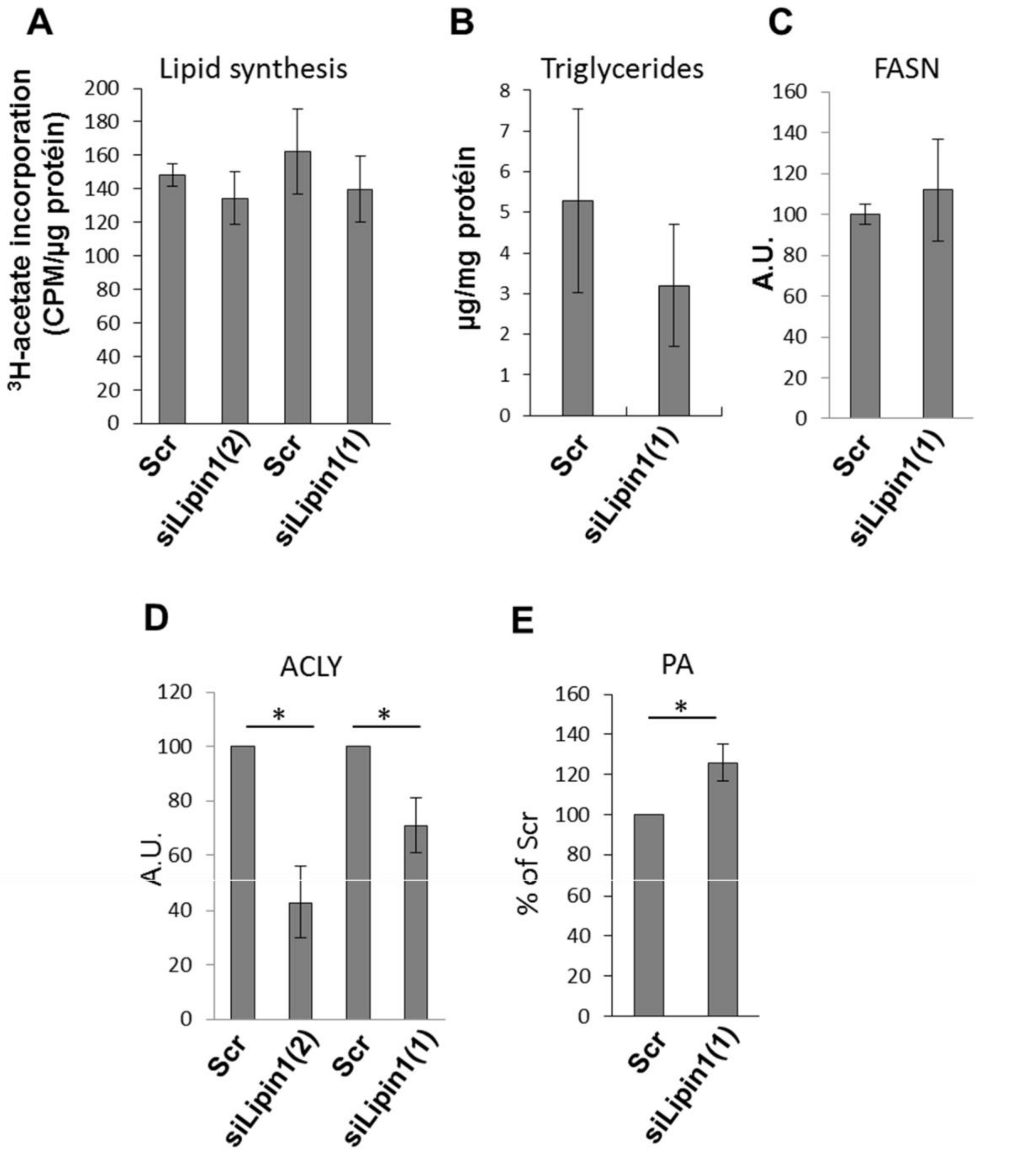


Supplemental Fig.6. Lipin-1 silencing does not significantly affect lipid synthesis, triglycerides content or Fatty Acid Synthase (FASN) expression but increased Phosphatidic Acid (PA) content. PC-3 cells were transfected with the indicated siRNA. (A) 24 h after transfection, cells were incubated with $^{3}$H-acetate for 24 h. Cells were then collected and processed for measuring the $^{3}$H-acetate incorporated into the lipid phase as described in "Material and Methods". The results are representative of three independent experiments. (B) 48 h after transfection, cells were collected, lipids extracted and triglycerides measured as described in "Materials and Methods". (C-D) Total RNA was extracted 48 h after transfection. Fatty Acid Synthase (FASN) (C) and ATP citrate lyase (ACLY) (D) mRNA were amplified by RT- PCR. Data are expressed in arbitrary units (A.U.) relatively to the control condition set up to 100. (E) 48 h after transfection, cells were processed for Phosphatidic Acid measurements as described in "Materials and Methods". The results of each graph are the mean$\pm$s.d. of three independent experiments. *$p<0.05$ ANOVA followed by Tukey-Kramer analysis.

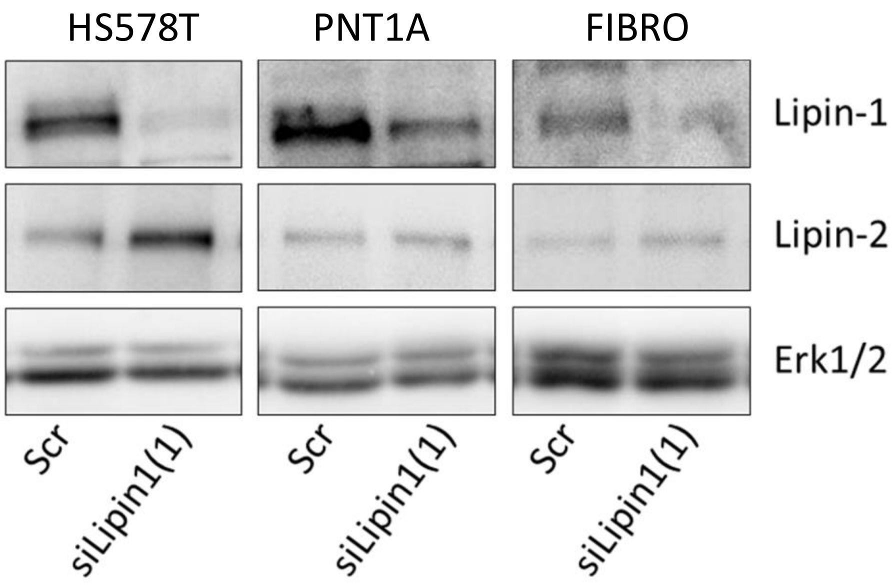


Supplemental Fig. 7. Compensatory regulations between lipin-1 and -2 in Hs578T cells but not in PNT1A cells or fibroblasts. 48 h after transfection with the indicated siRNA, cells were lysed and analyzed by Western blotting with specific antibodies to lipin-1, lipin-2 and Erk1/2. Representative blots are shown.

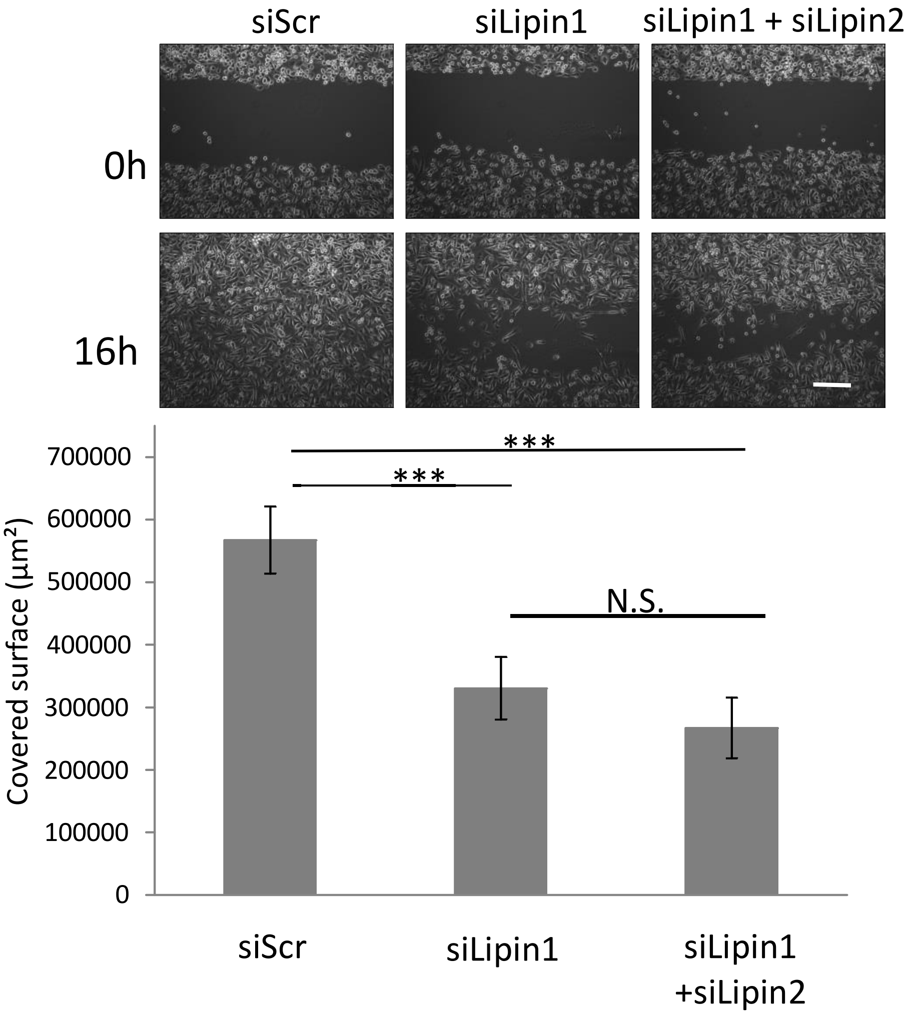


Supplemental Fig.8. The inhibition of migration mediated by lipin-1 silencing is not altered following co-silencing lipin-2. Immediately after transfection with 20 nM of a control siRNA, 20 nM of the first siRNA targeting lipin-1 (siLipin1) or 20 nM of the first siRNA targeting lipin-1+20 nM of an siRNA targeting lipin-2 (siLipin1+siLipin2) cells were processed for the wound healing assay as described in "Materials and Methods". Representative phase contrast microscopy photographs were taken immediately after releasing the insert (0h) and 16 hours later (16h). Bar = 250 µm. N.S.: not significant ***$p<0.001$ ANOVA followed by Tukey-Kramer analysis. The graphs summarize the results of three independent experiments.

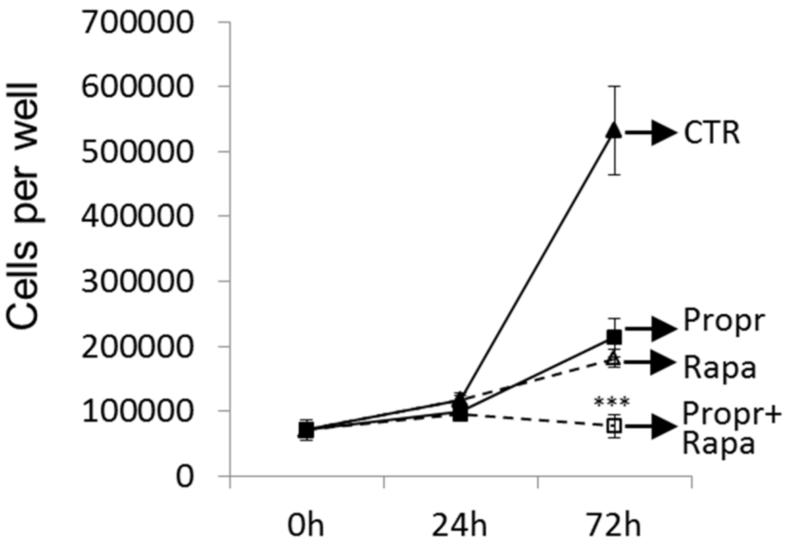


Supplemental Fig. 9. Potentiation of the anti-proliferative effect of rapamycin by pharmacological inhibition of lipin-1 as determined by direct cell counting. PC-3 cells seeded in 12-well plates were treated with 50 nM rapamycin (rapa) and/or 100 µM propranolol (propr). The number of cells in each well was measured as described in "Materials and Methods". *** $p < 0.001$ ANOVA followed by Tukey-Kramer analysis.

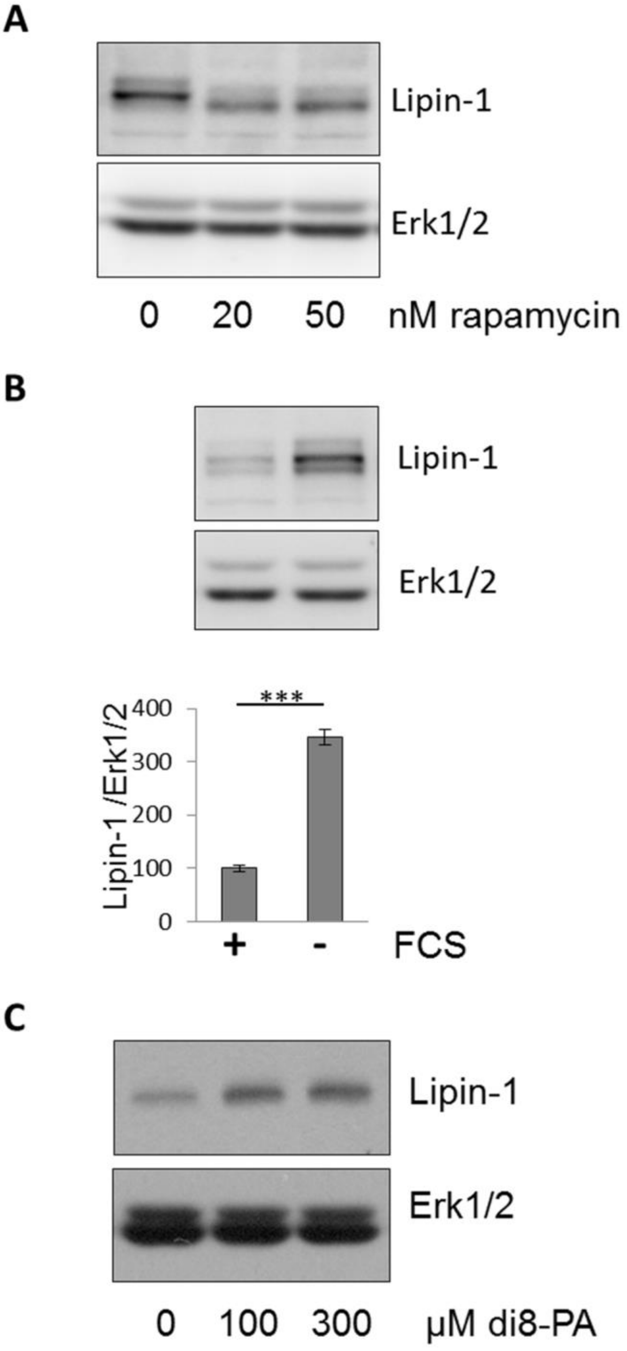


Supplemental Fig.10. Western blot analyses of PC-3 cells lysates with specific antibodies to Lipin-1 and Erk1/2. In (A), cells were cultured in presence of the indicated concentration of rapamycin or vehicle (0) during 24 h. The shift down of the band corresponding to lipin-1 suggests a decreased phosphorylation of the protein. In (B), cells were cultured with (+) or without (-) serum during 24 h. The graph below summarizes the results of three independent experiments expressed as mean±s.d. In (C), cells were cultured in presence of the indicated concentration of 1,2-Dioctanoyl-sn-glycerol 3-phosphate sodium salt (di8-PA) during 24 h. ***$p<0.001$ ANOVA followed by Tukey-Kramer analysis.
